# Melioidosis Vaccines (MeVa): Attitudes to vaccines, melioidosis and clinical trials in key stakeholders in Ubon Ratchathani, Thailand

**DOI:** 10.12688/wellcomeopenres.18383.1

**Published:** 2023-09-19

**Authors:** Napat Khirikoekkong, Supa-at Asarath, Jennifer Hill, Benjawan Wettana, Orathai Srisawang, Phaik Yeong Cheah, Susanna Dunachie, Parinya Chamnan

**Affiliations:** 1 Mahidol Oxford Tropical Medicine Research Unit, Bangkok, 10400, Thailand; 2 NIHR Oxford Biomedical Research Centre, Oxford University Hospitals Trust, Oxford, UK; 3 NDM Centre for Global Health Research, University of Oxford, Oxford, OX1 3SY, UK; 4 Department of Social Medicine, Sanpasitthiprasong Hospital, Ubon Ratchathani, 34000, Thailand; 5 The Ethox Centre, Nuffield Department of Population Health, University of Oxford, Oxford, UK; 6 Centre for Tropical Medicine & Global Health, Nuffield Department of Medicine, University of Oxford, Oxford, UK

**Keywords:** Melioidosis, vaccine attitude, vaccine perception, vaccine acceptance, qualitative, research participation

## Abstract

**Background:** Melioidosis is a bacterial infection which kills an estimated 89,000 people per year in tropical and sub-tropical regions, chiefly affecting the poorest. Diabetes is the primary risk factor, conferring a 12-fold increase in risk. Despite limited funding compared to other neglected tropical diseases, melioidosis vaccine development has generated several candidates for clinical development. CPS-CRM _197_/Hcp1 is a promising vaccine candidate developed at the University of Nevada, Reno which is due to enter a Phase I clinical trial in Oxford, UK in 2024. As we move closer to the possibility of field trials of a melioidosis vaccine, it is critical to work in parallel to understand perceptions toward a vaccine among those living where melioidosis rates are high. Reasons for vaccine acceptance versus hesitancy are complex, and include perceived risk of the target disease, concern about side effects, and above all trust in government, scientists, the pharmaceutical industry and other authorities.

**Methods:** We will carry out a qualitative study in Ubon Ratchathani, Thailand, an endemic region for melioidosis, as groundwork for a potential future melioidosis vaccine efficacy study, and in the longer-term vaccine introduction. This study seeks to explore knowledge and attitudes in three main areas; 1) melioidosis disease, 2) vaccines, and 3) participation in clinical vaccine trials. In-depth interviews and focus group discussions will take place in five participant groups of different risks and exposure to melioidosis. Purposive, convenience sampling will be used, also snowball sampling to reach some participant groups. Sample size will be based on participant’s experience, to inform the line of enquiries of study, or until data saturation, expecting 66–90 participants across all groups.

**Discussion:** The findings of this study will be written up and published in an open access journal, and will be valuable to inform future design of clinical trials as well as engagement and communications associated with future vaccine rollout.

## Introduction

### Melioidosis

The causative agent of melioidosis, bacterium *Burkholderia pseudomallei,* resides in wet soils and rice paddies, and infects through cuts in the skin and inhalation of aerosolised bacteria. The clinical spectrum of melioidosis is broad but in the majority of cases infection gives rise to severe illness which is then difficult to treat. Mortality rates are highly variable by region and often > 50% in low- and middle-income countries (LMICs). Melioidosis infection has several risk factors, the strongest of these being diabetes which increases the risk of infection 12-fold ^1, 2^. Other risk factors include older age, kidney or liver disease and excessive alcohol consumption ^3^. Hopes are high for the development of an effective vaccine ^4^; a melioidosis vaccine offering a good degree of protection delivered to diabetics is likely to be a cost-effective intervention ^5^.

### Melioidosis vaccine development

Several vaccine types have been explored in animal models for potential to prevent melioidosis, and numerous candidates have demonstrated some degree of protection ^6^. A promising subunit candidate vaccine has been developed at University of Nevada, Reno (CPS-CRM _197_/Hcp1) with plans for a Phase I clinical trial in Oxford, UK in 2024 to determine safety and immunogenicity in healthy adult volunteers with and without diabetes ^7^. This trial of a melioidosis vaccine in humans will be a landmark in the struggle to combat the devastating impact of this disease, and it is hoped will pave the way for the testing of other promising candidates. Providing the safety profile of the vaccine in humans is acceptable and immunogenicity is demonstrated, further studies will seek to replicate immunogenicity in populations in endemic regions, and assess vaccine efficacy.

### Site selection for melioidosis vaccine trials

Site selection for a phase II vaccine efficacy trial takes into account a range of factors, including the size of the population at risk, local incidence of the disease, and infrastructure to support clinical trial activities. Thailand is endemic for melioidosis, with a high incidence documented over several decades in the region of Ubon Ratchathani in Northeastern Thailand ^8– 10^. With established infrastructure to support research in the area, Ubon Ratchathani is a strong candidate site for a phase II melioidosis vaccine trial. Local community engagement with the research from an early stage is critical to the success of a clinical trial. The trial design must be appropriate to the local setting, and consider relevant cultural, religious, political and environmental factors. In Ubon Ratchathani, gaining insight into the perspectives of people who could be eligible to take part in a melioidosis vaccine trial on the topics of vaccination, melioidosis, and taking part in research, will help to identify the needs of potential volunteers.

### The intersection of vaccine acceptancy/hesitancy and cultural beliefs

Once a vaccine is licensed, widespread acceptance is crucial in order to fully realise the potential benefit in prevention of disease, and maximise vaccine cost effectiveness. Reasons for vaccine acceptance versus hesitancy are complex and vary in each context, they include perceived risk of target disease, concern about side effects, and above all trust in government, scientists, the pharmaceutical industry and other authorities. To date, qualitative studies have provided insight into public knowledge of coronavirus 2019 (COVID-19) and influenza, and attitudes towards vaccination against these infections in Thailand ^11– 13^. These studies uncovered factors connected to vaccine hesitancy, for example low education levels ^12^ and the presence of existing health conditions in older adults ^11^, and have identified social influence by family, peers and healthcare providers as a strong driver in decision-making around vaccination ^13^. Studies of attitudes toward COVID-19 vaccination in Thailand report relatively high rates of hesitancy to be vaccinated ^11, 12^, but connectedness of attitudes toward COVID-19 vaccination and vaccination against other diseases are to be explored. At the level of the whole country, a recent (2016) analysis suggests that feelings about vaccination in Thailand are amongst the most positive internationally, with attitudes towards the importance, safety, and efficacy of vaccination in general more favourable than on average across the 67 countries included ^14^. However just one country ranked higher in the proportion of respondents expressing a negative reaction to the statement ‘Vaccines are compatible with my religious beliefs’, highlighting that cultural and religious factors may be of particular importance in driving the willingness of adults in Thailand to consider undergoing vaccination.

## Aims

The aim of this study is to gather the perspectives of key population groups relevant to clinical trials and rollout of melioidosis vaccines, on the topics of melioidosis, taking part in clinical trials for melioidosis vaccines, and vaccination in general and against COVID-19 specifically. The study will explore beliefs, perceived risks, benefits and factors influencing position of vaccine acceptancy/hesitancy, among diabetes patients, patients’ relatives and persons with risk of exposure to melioidosis in Ubon Ratchathani.

The findings of this study will provide important insight into motivations for taking part in clinical trials as well as associated concerns, which will inform the feasibility of future trials and facilitate sensitivity to the local setting in the study design. Healthcare providers involved in promoting vaccination among individuals with a high risk of melioidosis will benefit from this information to design suitable interventions and treatment in the future.

## Methods

### Study site

This study will be carried out within the Ubon Ratchathani Province, Thailand, in four districts with high cases of melioidosis, while maintaining balance with districts with diabetic patients (Mueang, Lao Suea Kok, Samrong, and Warin Chamrap districts) ( Table 1).

**Table 1.** Districts and sub-districts for participant recruitment with data collection location.

Name of district	Name of sub-district	Location of interviews
Mueang district	Tha Wang Hin	Tha Wang Hin PCU’s meeting room
Mueang district	Rai Noi	Dong Saen Suk PCU’s meeting room
Lao Suea Kok district	Nong Bok	Ban Doon PCU’s meeting room
Samrong district	Samrong	Samrong PCU’s meeting room
Warin Chamrap district	Non Phueng	Thung Kasem PCU’s meeting room

Ubon Ratchathani is located in the lower part of Northeastern Thailand, bordering Cambodia and Laos PDR. The general topography of the province include flood plain (river levee, and back swamp), alluvial plain, peneplain, and piedmont. Two main rivers flow through the centre of the province towards the Mekong River. To support agricultural activities, there are many irrigation canals and sub-canals spread across the province. The rainy season is from May to end of October, marking the farming and growing season. The main occupation of the Ubon Ratchathani population is in the agricultural sector, also over half of total province area is for agricultural purposes (66.12% of total area), including paddy fields (47.85%).

### Study design

This study is an exploratory research study, utilizing qualitative research methodology in data collection, including in-depth interviews (IDIs) and focus group discussions (FGDs). Interview notes and field notes will be taken during or after data collection sessions to record any other observations an audio recorder is unable to capture. Participants in this study will be invited to share their thoughts in semi-structured interviews and/or focus group discussions, to approach intended outcomes

Questions in topic guides are designed to ascertain experience and levels of awareness of melioidosis, thoughts and opinions on undergoing vaccination and taking part in a melioidosis vaccine trial, as well as the basis for these. A topic of much relevance currently, attitudes towards vaccination against COVID-19 will also be explored as part of the discussion on vaccination in general.

### Study outcomes

**Primary outcome:** An account of the views and perceptions of diabetes patients in Ubon Ratchathani on the topics of melioidosis infection, vaccination, and taking part in clinical trials for melioidosis vaccines.

**Secondary outcomes:**

1) An account of views on vaccination in general including perceived benefits and risks, and the enabling factors and barriers. An account of views on COVID-19 vaccination specifically, the general feeling of the community regarding COVID-19 vaccination, and assembled concerns.

2) An account of knowledge and understanding of the cause, severity, diagnosis, and symptoms of melioidosis. An account of knowledge and understanding of melioidosis risk factors, and preventative measures for melioidosis including vaccination.

3) An account of attitudes towards taking part in clinical trials of melioidosis vaccines including the risks and benefits, and the nature and sources of concerns and drivers.

### Sampling technique, participant recruitment, sample size, and eligibility criteria

Purposive, convenience sampling techniques will be mainly used to identify research participants for this study, with occasional snowball sampling technique for specific participant groups. As a qualitative study, sample size will be based on experience of participants, to inform the line of enquiries of study, or until data saturation. Approximately 26 IDIs will be conducted, and more if needed. Approximately eight FGDs will take place, with between five and eight participants in each (between 40 and 64 FGD participants in total). Therefore, the total number of participants across both data collection methods is estimated to be between 66 to 90 ( Table 2). The study team will work closely with the Primary Care Unit within identified districts in order to cautiously identify potential participants.

**Table 2.** Group of participants by data collection methods.

Participants	Approximate numbers of In-depth Interviews (IDIs) participants	Approximate number of Focus Group Discussions (FGD, 5–8 participants in each group)
**Group 1 – Diabetes patients** a) 20–60 years old, and b) over 60 years old	5	2
**Group 2 – Melioidosis patients** a) 20–60 years old, and b) over 60 years old	5	2
**Group 3 – Villagers with livelihood or ** **occupation with risks of exposure to ** **melioidosis** Participants aged 20 and above	5	2
**Group 4 – Adult relatives of diabetes ** **or melioidosis patients** Adult relatives: child, spouse, niece or nephew (aged 20 and above) Or non-relative adult caregiver and main supporter aged 20 and above	5	2
**Group 5 – Key informants** Healthcare personnel aged 20 and above. healthcare worker at hospital, PCU, health centre; village health volunteer, local elders, village chief or respectful and knowledgeable persons	6	-
**Total**	26 participants	8 groups (estimated 40–64 participants)

### Recruitment criteria of each participant group

**Group 1 and 2** will consist of diabetes patients, and present or former melioidosis patients between a) 20–60 years old, and b) over 60 years old. They will be identified through existing records held at primary care units for diabetes clinic attendance, and existing records of melioidosis patients.

**Group 3** participants will be villagers living in the endemic area, with exposure risk to melioidosis from occupation or livelihood, and interest to take part in the study. The potential participants will be identified by local/area key persons, for example village chief, local healthcare workers, village health volunteers.

**Group 4** participants will be a caregiver or main supporter, relative or nonrelative, of a patient with diabetes or melioidosis, and will be identified by participants in groups 1 and 2. Researchers will contact potential group 4 participants only with the agreement from the corresponding participant of group 1 or 2.

**Group 5** participants will include healthcare personnel, local experts, community leaders including village chief, and elder with in-depth understanding of the study site context. The work role, or area of expertise, or experiences of healthcare personnel and staff would be taken into consideration.

Most participants will take part in a single interview, although if needed a follow up interview may be conducted. Permission for each follow-up interview will be sought from the participant. The choices and decisions of participant will be respected. For each case, the study team will contact participant via phone to seek their willingness and availability for follow-up interview.

 An alternative district will be identified by the Principal Investigator (PI) and study team should recruitment be unsuccessful at any of the sites outlined in Table 1. Listed interview locations may be changed to accommodate participants’ privacy, preference, and convenience. Venue rental costs will be covered by the study.

### Inclusion criteria

Participants for all groups aged 20 and above, with ability to provide written informed consent and able take part in the interviews.

### Exclusion criteria

Persons that are not able to provide written consent, unfit and unable to take part in the interviews.

### Inclusion of healthy volunteers

This study will recruit healthy volunteers, those apart from diabetes and melioidosis patients, who are villagers, and farmers with risk of exposure to melioidosis in their work or daily lives, to triangulate the views and perspectives on vaccine attitudes, and participation in clinical trials among villagers living in melioidosis endemic area. The study team will seek to minimise disruption to participant’s livelihoods in the arrangement of interview times and venues. 

### Inclusion of vulnerable participants

This study will include diabetes and melioidosis patients aged above 60 years old. Both diabetes and melioidosis are considered chronic conditions due to the need for prolonged treatment. Half of melioidosis patients have underlying diabetes, and the majority of patients diagnosed with diabetes are elderly. Including them in this study is important to ensure that perspectives of population groups at the greatest risk of melioidosis are represented.

### Withdrawal of participant criteria

All participants have the right to withdraw from the study at any point without having to provide reason. If data has been collected from a participant requesting to withdraw, they will be asked if the data collected can be used for analysis or not, and the participant’s decision will be respected. At any time, if a participant wishes to stop an interview the participant’s decision will be respected. Should a participant become or feel distress from taking part in this study and interview, consultation with PI and co-investigators will be conducted. This is to ensure that appropriate referral is arranged with participant’s knowledge, and consent is obtained for any additional supports in area of need(s).

Participation of this study is purely voluntary. There will be no negative consequences should they decide to stop their participation or withdraw from the study after they have decided to participate.

### Data collection

This study will use two types of interviews (IDI and FGD). The discussion will be led by study team members trained and experienced in these interview approaches, and in accordance with a topic guide (see *Extended data* ^15^). Discussion topics will remain within scope of the objectives. Interviews (both IDI and FGD) will be carried out in Thai, and in Northeastern Thai dialect when necessary. IDIs will be conducted in a private room at a Primary Care Unit (PCU) or in an open space where the participant(s) feel comfortable to freely express their views

All participants will be approached face-to-face, should it be allowed, with consideration of COVID-19 measures and safety of participants, and restrictions for each area, if relevant. Should a face-to-face contact not be allowed or suitable, participants will be contacted by telephone to inform them about the study, and to seek their agreement to participate.

Persons making contacts will be the co-investigators and researchers of this study, Napat Khirikoekkong and Supa-at Asarath, with close support from Benjawan Wettana and Orathai Srisawang.

### In-depth interviews (IDIs)

Each interview is to last between 45 minutes and 1.5 hour. Follow-up interviews may take place with prior permission from the participant should additional information be required.

IDI interviews will be conducted through a telephone call should a face-to-face interview not be possible (e.g., due to COVID-19 restrictions, if relevant) or not preferred by the participant. Consent will be obtained before interview date.

### Focus group discussions (FGDs)

FGDs will last between 1 and 1.5 hours, with participants to take a short break on request.

Focus group discussions are best conducted in-person to stimulate discussion from participants with diverse backgrounds and views. If necessary, group sizes will be adjusted for compliance with COVID-19 measures, if relevant. Consent will be obtained before interview date to confirm participant’s interest in taking part in the study and interview. The venue will be chosen to meet the requirement of group activity with strict observation of physical distancing, body temperature screening and mask wearing throughout the interview. Researchers and participants involved in FGD must present a negative COVID-19 test result e.g., antigen self-test kit (ATK). The test should be taken in the 24 hours before the scheduled interview, with consideration and compliance of recommended measures from provincial and district public health office.

All interviews (both IDIs and FGDs) will be audio recorded to ensure that discussions are properly captured.

### Compensation

Compensation will be provided to participants taking part in IDIs and FGDs. Each participant will receive THB500 ( *est. GBP12*), for one interview lasting between 45 minutes to 1.5 hour to compensate for their time to take part, traveling fees, and loss of income for the period which they took part in the interview. Participants will be provided with refreshments at the interview venue.

### Patient and public involvement

We will engage with patients and members of the public in a Patient and Public Involvement session. The project including topic guide for interview and focus group discussion developed for IDIs and FGDs will be discussed with 16 invited participants, including melioidosis patients, diabetes patients, care givers to patients, people living in endemic areas, and healthcare professionals and key informants including village health volunteers, PCU staff, and village chief. The same individuals will not be approached to take part in the main study. Their suggestions on both topic guides or any discussion raised from the session will be taken into account to make the topic guides more applicable and suitable to context, with participants outside of Ubon Ratchathani town.

## Data management

### Data recording and record keeping

Participants will be notified that their interview will be recorded during the consent process, and be reminded again when the data collection session is about to commence. All data will be securely stored on encrypted external hard-disks and password-protected laptops. Audio recordings will be transferred from recorders to a laptop, and recorders erased daily to ensure that data of the participants remains confidential.

### Data processing and analysis

The data produced will consist of audio recordings, transcripts, and field notes. All audio files will be transcribed and translated verbatim into English. The interview and focus group discussion transcripts, including relevant text files, will be managed using NVivo (released in March 2020) ^16^. Audio files will be kept until transcribed and analysed, transcribed interviews files will be kept in secure servers with a proper backup system. The study team will de-identify participants’ information to maintain confidentiality.

The analysis will include content analysis, narrative analysis, and thematic analysis, following with open coding to find and group the emerging themes. The coding will begin as early as the first few transcriptions are ready. Any language ambiguities, likely from Northeastern dialects, will be discussed thoroughly with local co-investigators who speak and understand the dialects. Interview notes and field notes will also be taken into consideration for formal content analysis, which could shape the data and validate the data collected from that specific interview or focus group discussion. Axial coding, or second passing coding will be done with the wider team which include the main investigators.

### Data protection

The University of Oxford is the data controller with respect to participants’ personal data, and as such will determine how the personal data is used in the study. The University will process the personal data for the purpose of the research outlined above. Research is a task that is performed in the public interest.

### Data availability and access to data

Access to data which are recorded audio files, interview notes, field notes and interview transcripts and photographs will be granted to authorised representatives from the sponsor, host institution, Ethics Committees, regulatory authorities for monitoring and/or audit of the study to ensure compliance with regulations. Data collected for this study will be under the custodianship of Mahidol Oxford Tropical Medicine Research Unit. The data will be de-identified. De-identified data may be shared with other groups of researchers upon reasonable request, in accordance with the current MORU Data Sharing Policy.

## Ethics and dissemination

**Research ethics:** This is a minimal risk study posing very low risk. There are no medical or behavioural interventions to participants. The main ethical issues are related to privacy and confidentiality. The interview and focus group discussion venues will be private and comfortable spaces for all participants. Care will be taken to maintain privacy during the audio recording of data collection processes, with both individuals and group participants.

**Research ethical approval:** This study has been granted ethical approval from the Oxford Tropical Research Ethics Committee (OxTREC), reference number: 533-22 (dated 19 July 2022), and the Ethics Committee in Human Research of Sanpasitthiprasong Hospital, study identifier code 040/65C (dated 21 December 2022).

**Consent:** Main co-investigators (Khirikoekkong N., and Asarath S.) are responsible for seeking and obtaining written informed consent and will lead this process with the assistance of local co-investigators (Wettana B., and Srisawang O.). Local co-investigators will provide necessary interpretation between Thai and local dialect Thai. No assent will be use in this study.

**Confidentiality:** Participants will be informed in prior to data collection that the session will be audio recorded and transcribed. The transcripts will be de-identified for publication and for data sharing, if any. All study team members will adhere and ensure that participants’ confidentiality is well maintained.

Participating in an interview may result in loss of privacy, information provided during interviews will be recoded but names or any personal information will not be used in any reports and all personal information will be de-identified to protect participant’s privacy.

Participant can skip or not answer questions that they do not feel comfortable answering.

Any mention of any persons during an interview will be anonymized to protect their identity and privacy.

**Dissemination policy:** The results of this study will be published in an open access journal. The findings will inform the feasibility of future vaccine studies, as well as identify perceived risks, benefits, or other ethical and biological concerns regarding vaccine studies, clinical trials, and involvement of human participants. The dissemination of this research will support the design of future research to be more responsive and sensitive to the local context and setting.

## Study status

Data collection started on 20th February 2023 and is on-going. As of the 14 ^th^ May 2023 the number of participants recruited across all groups is 32.

## Data Availability

No underlying data are associated with this article. Zenodo: Topic Guide for In-depth Interview and Focus Group Discussion for MeVa. https://doi.org/10.5281/zenodo.7691314 ^15^. This project contains the following extended data: 20221204_MeVa_Topic Guide IDI_FGD_V2_ENG.docx Data are available under the terms of the Creative Commons Attribution 4.0 International license (CC-BY 4.0).
