# Supplementary material for: Melioidosis Vaccines (MeVa): Attitudes to vaccines, melioidosis and clinical trials in key stakeholders in Ubon Ratchathani, Thailand
Source: Wellcome Open Res. 2023 Sep 19;8:413. [Version 1] doi: 10.12688/wellcomeopenres.18383.1 (PMC10646340; doi:10.12688/wellcomeopenres.18383.1)
Supplement: Peer review history

# Reviewer report: Reviewer response for version 1

**DOI:** 10.21956/wellcomeopenres.20377.r67432

**Published:** 2023-11-14

**Authors:** Ella Meumann

**Affiliations:** 1 Global and Tropical Health Division, Menzies School of Health Research, Charles Darwin University, Casuarina, Northern Territory, Australia

Thank you for the opportunity to review this manuscript outlining the methodology for a qualitative research study on the attitudes of people in northeastern Thailand towards vaccines, melioidosis, and clinical trials.

 The rationale for this work is clearly articulated. A phase I clinical trial for a subunit vaccine for melioidosis will commence in the UK this year, with phase II efficacy studies in endemic areas likely to follow. Importantly, design of such an efficacy study should be appropriate to the local setting. This study will help to identify the needs of potential volunteers in an endemic area which is potentially suitable for such a trial.

 The study design is appropriate. The study will include 66-90 participants comprising people living with diabetes (a major risk factor for melioidosis), people with previous melioidosis, support persons for the former two groups, people at risk of melioidosis exposure, and experts and community leaders. In depth interviews and focus group discussions will be conducted to investigate knowledge, understanding and attitudes towards vaccination (for melioidosis, for COVID-19, and in general), melioidosis, and clinical trials for a melioidosis vaccine. The questions included in the Topic Guide (Extended data), and the approach to recruitment, consent, participant withdrawal, data management, privacy and confidentiality are appropriate.

 In the Introduction there is a section outlining factors associated with vaccine hesitancy - including trust in authorities, education levels, and cultural and religious factors. It isn't clear from the manuscript whether or how these factors will be explored in the study. It also isn't clear whether the analysis will look at whether there are particular trends among the different groups of study participants outlined above, and whether attitudes vary among people with different demographics such as age, gender, occupation, or region. I would recommend that this be clarified.

 Overall, the planned research forms an important component of melioidosis vaccine phase II clinical trial design, and will ensure that such a study is most likely to meet the needs of trial participants - and ultimately those who stand to benefit most from a vaccine to prevent melioidosis.

Is the study design appropriate for the research question?

Yes

Is the rationale for, and objectives of, the study clearly described?

Yes

Are sufficient details of the methods provided to allow replication by others?

Yes

Are the datasets clearly presented in a useable and accessible format?

Not applicable

Reviewer Expertise:

Genomic epidemiology of infectious diseases, including melioidosis

I confirm that I have read this submission and believe that I have an appropriate level of expertise to confirm that it is of an acceptable scientific standard.

# Reviewer report: Reviewer response for version 1

**DOI:** 10.21956/wellcomeopenres.20377.r67431

**Published:** 2023-10-10

**Authors:** Oluwatosin B. Adekeye

**Affiliations:** 1 Department of Psychiatry, Ahmadu Bello University Teaching Hospital Shika, Zaria, Nigeria

The study aims to gather the perspectives of key population groups relevant to clinical trials and roll out of melioidosis vaccines. To explore the beliefs, perceived risks, benefits, and factors influencing acceptance among diabetes patients. 

 INTRODUCTION - This section contains adequate background to the study. Still, it would also benefit from a mention of reasoned action as attitudes and subjective norms contribute to intention and behavior (acceptance of vaccine). 

 The last sentence of the Aim could include designing a context-specific intervention in close partnership with the community. 

 STUDY DESIGN - this is adequate. 

 PARTICIPANTS - Exclusion criteria based on not being able to provide written consent could be re-considered as verbal consent and thumb print is also an option for consent.

 Not much mention is made of in the study of consideration of gender. This is important as attitudes can be perceived from a gendered perspective. It would also be important in the analysis since this is a qualitative study to look at gender. 

 GROUP 4 - the participants in this group should read primary care giver.

 GROUP 5 - this should also take into consideration the role of community leaders in influencing attitude change and being co-participants in designing the context specific intervention. 

 CONSENT - as part of eliciting consent, also include Participant Information Sheets that provide participants with all the information required for the study to enable them provide adequate consent.

Is the study design appropriate for the research question?

Yes

Is the rationale for, and objectives of, the study clearly described?

Yes

Are sufficient details of the methods provided to allow replication by others?

Yes

Are the datasets clearly presented in a useable and accessible format?

Not applicable

Reviewer Expertise:

Qualitative research, Clinical trials

I confirm that I have read this submission and believe that I have an appropriate level of expertise to confirm that it is of an acceptable scientific standard.
